# Study of high–low KPFM on a pn-patterned Si surface

**DOI:** 10.1093/jmicro/dfab055

**Published:** 2022-01-11

**Authors:** Ryo Izumi, Yan Jun Li, Yoshitaka Naitoh, Yasuhiro Sugawara

**Affiliations:** Department of Applied Physics, Graduate School of Engineering, Osaka University, 2-1 Yamadaoka, Suita, Osaka 565-0871, Japan; Department of Applied Physics, Graduate School of Engineering, Osaka University, 2-1 Yamadaoka, Suita, Osaka 565-0871, Japan; Department of Applied Physics, Graduate School of Engineering, Osaka University, 2-1 Yamadaoka, Suita, Osaka 565-0871, Japan; Department of Applied Physics, Graduate School of Engineering, Osaka University, 2-1 Yamadaoka, Suita, Osaka 565-0871, Japan

**Keywords:** heterodyne, FM-KPFM, CPD, surface potential, Si, band bending

## Abstract

Comparative measurements between frequency modulation Kelvin probe force microscopy (FM-KPFM) using low frequency bias voltage and heterodyne FM-KPFM using high frequency bias voltage were performed on the surface potential measurement. A silicon substrate patterned with p- and n-type impurities was used as a quantitative sample. The multi-pass scanning method in the measurements of FM-KPFM and heterodyne FM-KPFM was used to eliminate the effect of the tip–sample distance dependence. The measured surface potentials become lower in the order of the p-type region, n-type region and n^+^-type region by both FM-KPFM and heterodyne FM-KPFM, which are in good agreement with the order of the work functions of the pn-patterned Si sample. We observed the difference in the surface potentials due to the surface band bending measured by FM-KPFM and heterodyne FM-KPFM. The difference is due to the fact that the charge transfer between the surface and bulk levels may or may not respond to AC bias voltage.

## Introduction

The surface and interface states of semiconductors have a considerable effect on the reliability of semiconductor devices such as metal-oxide-semiconductor field-effect transistors (MOS-FETs) [1–4]. Since the surface states of semiconductors are related to their surface potentials, information on the surface potentials of semiconductors is important because it provides important insights into the electrical properties of the surface. To understand and control the surface states of semiconductor devices, it is important to measure the surface states of semiconductors, especially the surface potential, with high spatial resolution.

Kelvin probe force microscopy (KPFM) is a technique for measuring the surface potential of a sample with high spatial resolution [5–10]. KPFM is based on atomic force microscopy (AFM) [11–13]. The underlying principle of KPFM is that the contact potential difference (CPD) between the tip and the sample surface is detected from the change in the resonance frequency or amplitude of the cantilever by applying an AC bias voltage [7,14]. The AC bias voltage modulates the electrostatic interaction force between the tip and the sample. A DC bias voltage is used to nullify the average electrical force related to the CPD. The results of surface potential measurements of metals [15], semiconductors [8,9,16–20] and insulators [21,22] by KPFM have been reported.

It has been reported that the results of KPFM reflect the condition of surface states. According to Ref. [14], increasing the concentration of impurities in a semiconductor causes band bending near the semiconductor surface; as a result, the CPD values deviates from those expected from the bulk states. Therefore, the CPD values measured by KPFM are affected by both the surface and bulk states. Separating bulk and surface information is important for understanding the electronic states of semiconductor surfaces and bulks.

To distinguish the information of the surface state from that of the bulk state in KPFM, it is very effective to use the frequency dependence of the AC bias voltage. This idea can be explained as follows. When an AC bias voltage is applied between the probe and the surface in KPFM, the Fermi level of the tip changes up and down with respect to the surface and bulk Fermi levels. There is a limit of the response rate of electron emission from the surface state to the bulk state [1,23]. As a result, the electron transfer between the surface state and the bulk state is strongly influenced by the frequency of AC bias voltage. That is, at frequencies lower than the cutoff frequency (*f*c) (<100 kHz), which is determined by the rate of charge capture and emission at the surface state, charge transfer occurs between the surface and bulk states, resulting in an up and down band bending [1,23]. On the other hand, at frequencies higher than fc (> several 100 kHz), charge transfer between the surface state and bulk state is less likely to occur, resulting in a slower response of the surface state and less change in band bending.

In KPFM, three major measurement modes have been used [7]: frequency modulation KPFM (FM-KPFM), amplitude modulation KPFM (AM-KPFM) and heterodyne AM-KPFM. In FM-KPFM, an AC bias voltage at the frequency (< 1 kHz) within the bandwidth of the phase-locked loop (PLL) is applied, and the modulation component of the frequency shift of the cantilever is used to measure the CPD values. In AM-KPFM, an AC bias voltage near/at the first (or second) resonance frequency of the cantilever is applied, and the cantilever deflection at the first (or second) resonance frequency is used to measure the CPD values. In heterodyne AM-KPFM, an AC bias voltage at the frequency of the difference between the first and second resonance frequencies is applied, and the cantilever deflection at the second (or first) resonance frequency is used to measure the CPD values. Thus, FM-KPFM uses a low-frequency AC bias voltage, while AM-KPFM and heterodyne AM-KPFM use a high-frequency AC bias voltage. FM-KPFM and AM-KPFM (Heterodyne AM-KPFM) use different force detection modes, FM mode and AM mode, respectively, and it is difficult to switch between the two force detection modes, making it difficult to perform low and high frequency KPFM measurements at the same position on the sample surface.

Recently, we proposed a new KPFM, the heterodyne FM-KPFM, which uses the FM mode as the force detection mode [18]. This method is based on the heterodyne effect of cantilever vibration and electrostatic force and allows the use of high frequency AC bias without increasing the bandwidth of the cantilever deflection sensor, with only minor changes to the FM-KPFM configuration. By alternating between FM-KPFM and heterodyne FM-KPFM using low and high frequency AC bias voltages, respectively, we have demonstrated that we can separate the surface state information from the bulk state information of reduced TiO_2_(110) samples [18]. Hereafter, we refer to this method as high–low KPFM (HL-KPFM). In the reduced TiO_2_ sample, the effectiveness of HL-KPFM has not been fully discussed because the density of oxygen defects is unknown and the carrier concentration is also unknown; therefore, the use of a sample that can be discussed more quantitatively has been desired.

In this study, to demonstrate the effectiveness of HL-KPFM on the surface potential measurement, we performed comparative study between FM-KPFM measurement and heterodyne FM-KPFM measurement using a silicon substrate patterned with p- and n-type impurities as a quantitative sample. The measured surface potentials become lower in the order of the p-type region, n-type region and n^+^-type region by both FM-KPFM and heterodyne FM-KPFM, which are in good agreement with the order of the work functions of the pn-patterned Si sample. We observed the difference in the surface potentials measured by FM-KPFM and heterodyne FM-KPFM, which is due to the surface band bending.

## Theory

### FM-KPFM

First, we explain the operation principle of FM-KPFM. When bias voltage *V* is applied between the tip and the sample, the electrostatic force between the tip and the sample can be expressed by considering the tip–sample system as capacitor and can be written as follows:

(1) \begin{equation*}\begin{matrix} {{{\bf{\it{F}}}_{{\bf{ele}}}} = {1 \over 2}{{\partial {\bf{\it{C}}}} \over {\partial {\bf{\it{z}}}}}{{\left( {{\bf{\it{V}}} - {{\bf{\it{V}}}_{{\bf{CPD}}}}} \right)}^2}} \\ \end{matrix} \end{equation*}

where *C, z* and *V*_CPD_ are the capacitance, the distance and the CPD between the tip and the sample, respectively. Here, the electrostatic force is always attractive interaction because ∂*C* /∂*z* <0. (Denominated *z*-direction, electrostatic force perpendicular to the sample surface.) In FM-KPFM, *V *= *V*_DC_ +*V*_AC_ cos*ω*_m_*t* is applied, where *V*_DC_ is the DC bias voltage, and *V*_AC_ and *ω*_m_ are the amplitude and the modulation frequency of the AC bias voltage, respectively. By expanding the equation, we can show that the electrostatic force contains the component of modulation frequency *ω*_m_ as follows:

(2) \begin{equation*}\begin{matrix} {{{\bf{\it{F}}}_{{\bf{ele}}}}\left( {{{\bf{\it{\omega }}}_{\bf{m}}}} \right) = {{\partial {\bf{\it{C}}}} \over {\partial {\bf{\it{z}}}}}{{\bf{\it{V}}}_{{\bf{AC}}}}\left( {{{\bf{\it{V}}}_{{\bf{DC}}}} - {{\bf{\it{V}}}_{{\bf{CPD}}}}} \right)} \\ \end{matrix} \end{equation*}

In frequency modulation atomic force microscopy (FM-AFM), the modulation component of the frequency shift ***△****f* (*ω_m_*) due to the electrostatic force *F*_ele_ (*ω_m_*) is expressed by the following equation:

(3) \begin{equation*}\begin{matrix} {\Delta f\left( {{{\bf{\it{\omega }}}_{\bf{m}}}} \right) \simeq {{{{\bf{\it{f}}}_0}} \over {\bf{\it{k}}}}{{{\partial ^2}{\bf{\it{C}}}} \over {\partial {{\bf{\it{z}}}^2}}}{{\bf{\it{V}}}_{{\bf{AC}}}}\left( {{{\bf{\it{V}}}_{{\bf{DC}}}} - {{\bf{\it{V}}}_{{\bf{CPD}}}}} \right)} \\ \end{matrix} \end{equation*}

where *f*_0_ (=*ω*_0_/2π) and *k* are the resonance frequency and the effective spring constant of the cantilever, respectively. In KPFM, the DC bias voltage is feedback-controlled so that the *ω*_m_ component of the frequency shift ***△****f* (*ω*_m_) becomes zero during scanning of the sample surface. The *V*_CPD_ can be measured by recording the DC bias voltage when ***△****f* (*ω*_m_) becomes zero by feedback control.

**Fig. 1.**
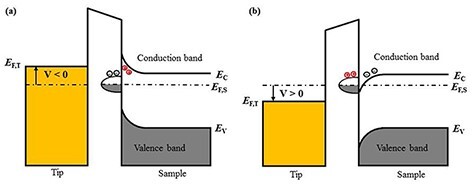
Schematic diagrams of field-effect-induced (lower frequency of AC bias voltage) band bending on n-type semiconductor with surface states and carriers transition between the surface state and bulk state. (a) *V* < 0 and (b) *V* > 0. Here, the surface-state-induced band bending is included and it shows the equilibrium between n-type bulk and its surface. (For simplicity, the work functions of the tip and the semiconductor are assumed to be the same.)

Note that in FM-KPFM, the modulation frequency *ω*_m_ is less than 2 kHz because the signal-to-noise ratio of the modulation component of the frequency shift ***△****f* (*ω*_m_) is degraded with increasing *ω*_m_ [24]. Here, we consider the carrier transfer between the accepter-like surface state (the dangling bond states) and bulk states, taking the n-type semiconductor as an example. In this case, electrons move so that the Fermi level of the surface level is equal to the bulk Fermi level, the surface level is negatively charged, the subsurface is positively charged, and the band bends upward at the surface, as shown in Fig. 1. When an AC bias voltage is applied between the tip and the surface, the Fermi level of the tip moves up or down with respect to the surface-state level. At frequencies *ω*_m_ lower than the cutoff frequency (*f*_c_) (<100 kHz), which is determined by the rate of charge capture and emission at surface states [1,23], the charge transfer between the surface states and bulk states easily occurs, and hence the band bending changes upwardly and downwardly. As a result, the *V*_CPD_ measured by FM-KPFM using low frequency AC bias voltage contains information on both the bulk Fermi level and the surface band bending.

### Heterodyne FM-KPFM

**Fig. 2.**
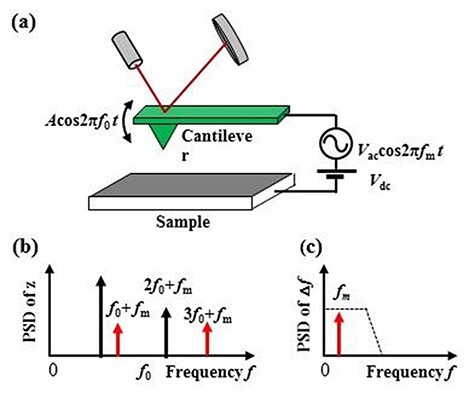
Schematic view of electrostatic interaction between tip and sample and PSD as function of frequency in heterodyne FM-KPFM. (a) Electrostatic interaction between tip and sample by applying *V*_DC_ and *V*_AC_ with frequency 2*f*_0_  *+f*_m_. (b) PSD of deflection *z* of frequency. (c) PSD of frequency shift Δ*f* as a function of frequency.

Next, we explain the theory of heterodyne FM-KPFM. The general setup of which is shown in Fig. 2. We consider the case where the cantilever is vibrating with amplitude *A* and angular frequency *ω*_0_. We can express the position of the tip of the cantilever as *z =* *z*_0_ +_ _*A* cos*ω*_0_*t*, where *z*_0_ is the mean distance between the tip and the surface. The capacitance between the tip and the sample depends on the distance between the tip and the sample. As a result, the capacitance voltage gradient ∂*C/*∂z can be expanded as

(4) \begin{equation*}\begin{matrix} {{{\partial {\bf{\it{C}}}\left( {\bf{\it{z}}} \right)} \over {\partial {\bf{\it{z}}}}} \simeq {{\partial {\bf{\it{C}}}\left( {{{\bf{\it{z}}}_0}} \right)} \over {\partial {\bf{\it{z}}}}} + {{{\partial ^2}{\bf{\it{C}}}\left( {{{\bf{\it{z}}}_0}} \right)} \over {\partial {{\bf{\it{z}}}^2}}}A\cos {{\bf{\it{\omega }}}_0}{\bf{\it{t}}}} \\ \end{matrix} \end{equation*}

By substituting Eq. (4) into Eq. (1), we can write the electrostatic force as

(5) \begin{equation*}\begin{matrix} {{{\bf{\it{F}}}_{{\bf{ele}}}} \simeq {1 \over 2}{{\partial {\bf{\it{C}}}\left( {{{\bf{\it{z}}}_0}} \right)} \over {\partial {\bf{\it{z}}}}}{{\left( {{\bf{\it{V}}} - {{\bf{\it{V}}}_{{\bf{CPD}}}}} \right)}^2} + {1 \over 2}{{{\partial ^2}{\bf{\it{C}}}\left( {{{\bf{\it{z}}}_0}} \right)} \over {\partial {{\bf{\it{z}}}^2}}}{{\left( {{\bf{\it{V}}} - {{\bf{\it{V}}}_{{\bf{CPD}}}}} \right)}^2}A\cos {{\bf{\it{\omega }}}_0}{\bf{\it{t}}}} \\ \end{matrix} \end{equation*}

In heterodyne FM-KPFM, the following bias voltage is applied between the tip and the sample:

(6) \begin{equation*}\begin{matrix} {V = {{\bf{\it{V}}}_{{\bf{DC}}}} + {{\bf{\it{V}}}_{{\bf{AC}}}}\cos \left( {2{{\bf{\it{\omega }}}_0} + {{\bf{\it{\omega }}}_{\bf{m}}}} \right){\bf{\it{t}}}} \\ \end{matrix} \end{equation*}

By expanding the second term of Eq. (5), one can see that the *ω*_0_  *+ω*_m_ component of the electrostatic force is included:

(7) \begin{equation*}\begin{matrix} {{{\bf{\it{F}}}_{{\bf{ele}}}}\left( {{{\bf{\omega }}_0} + {{\bf{\it{\omega }}}_{\bf{m}}}} \right) = {1 \over 2}{{{\partial^2}{\bf{\it{C}}}\left( {{{\bf{\it{z}}}_0}} \right)} \over {\partial {{\bf{\it{z}}}^2}}}{{\bf{\it{V}}}_{{\bf{AC}}}}\left( {{{\bf{\it{V}}}_{{\bf{DC}}}} - {{\bf{\it{V}}}_{{\bf{CPD}}}}} \right)A} \\ \end{matrix} \end{equation*}

Now, we consider the frequency shift formula:

(8) \begin{equation*}\begin{matrix} {\Delta f = - {{{\bf{\it{f}}}_0^{\,2}} \over {{\bf{\it{kA}}}}}\mathop \smallint \limits_0^{\bf{\it{T}}} {{\bf{\it{F}}}_{{\bf{ele}}}}\left( {\bf{\it{t}}} \right)\cos {{\bf{\it{\omega }}}_0}{\bf{\it{tdt}}}} \\ \end{matrix} \end{equation*}

As a result of the electrostatic force *F*_ele_ (*ω*_0_ + *ω*_m_), it can be seen that frequency shift ***△****f* (*ω*_m_) measured in FM mode is expressed as

(9) \begin{equation*}\begin{matrix} {\Delta f\left( {{{\bf{\it{\omega }}}_{\bf{m}}}} \right) = {{{{\bf{\it{f}}}_0}} \over {2{\bf{\it{k}}}}}{{{\partial ^2}{\bf{\it{C}}}\left( {{{\bf{\it{z}}}_0}} \right)} \over {\partial {{\bf{\it{z}}}^2}}}{{\bf{\it{V}}}_{{\bf{AC}}}}\left( {{{\bf{\it{V}}}_{{\bf{DC}}}} - {{\bf{\it{V}}}_{{\bf{CPD}}}}} \right)} \\ \end{matrix} \end{equation*}

In heterodyne FM-KPFM, as well as FM-KPFM, the *V*_CPD_ can be measured by recording *V*_DC_ when ***△****f* (*ω*_m_) becomes zero by feedback control. In heterodyne FM-KPFM, the AC bias voltage with high frequency is applicable. When frequency 2*f*_0_ + *f*_m_ is higher than fc (>several 100 kHz), the charge transfer between the surface states and the bulk states does not occur, and hence the band bending does not change owing to the slow response of the surface state. As a result, the *V*_CPD_ measured by heterodyne FM-KPFM using high frequency AC bias voltage mainly contains information on the bulk Fermi level.

## Experimental details

**Fig. 3.**
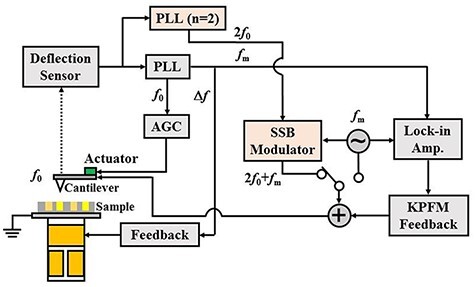
Block diagram of FM-KPFM and heterodyne FM-KPFM setup. Here, FM-KPFM is shown in grey and heterodyne FM-KPFM in pink. Note that an AC signal of cos2*ω*_0_*t* is generated by PLL (*n* = 2, the second harmonic mode). An AC bias signal of cos(2*ω*_0_ + *ω*_m_)*t* is generated by the SSB modulator. For more details, see Ref. [19].

Figure 3 shows the block diagram of FM-KPFM and heterodyne FM-KPFM combined with FM-AFM. Here, FM method is used to detect the force interaction between the tip and the sample. The frequency shift of cantilever ***△****f* is detected by using PLL circuit (Zurich Instruments: HF2PLL) by controlling oscillating amplitude *A* of the cantilever to be constant. Topographic images of the sample are obtained by controlling the distance between the tip and the sample so that frequency shift ***△****f* is constant. In FM-KPFM, an AC bias voltage $\cos {\omega _{\rm{m}}}t$ is generated by the local oscillator. In heterodyne FM-KPFM, an AC signal cos2*ω*_0_t is generated by PLL under the second harmonic mode (*n* = 2) synchronized with the cantilever oscillation. An AC bias voltage of cos(2*ω*_0_ + *ω*_m_)*t* is generated by mixing the signal of cos2*ω*_0_*t* with that of cos*ω*_m_*t* using a single sideband (SSB) modulator. In both FM-KPFM and heterodyne FM-KPFM, the *ω*_m_ component of frequency shift Δ*f*_m_ is measured with a lock-in amplifier and used to regulate the DC bias voltage *V*_dc_. KPFM images are obtained by regulating V_dc_ to nullify *Δf*_m_. The experiment was performed in a vacuum environment at room temperature using a commercially available atomic force microscope (JEOL: JSPM-4210).

**Fig. 4.**
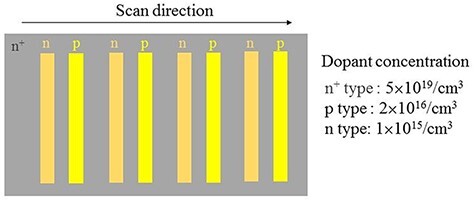
Model of the pn-patterned Si substrate. The scan direction of surface imaging was performed as shown by arrow.

As a sample, a silicon substrate regularly doped with p- and n-type impurities was used. Figure 4 shows the pn-patterned Si substrate. The dopant pattern on the sample surface was formed by ion implantation. The p-type region was formed by implanting BF_2_ ions with a dose of 7.0 × 10^12^/cm^2^ at 70 keV into a phosphorus-doped n-type Si substrate with a dopant concentration of 1 × 10^15^/cm^3^. The n+-type region was formed by implanting As ions with a dose of 3.0 × 10^12^/cm^2^ at 120 keV into the Si substrate. Impurity concentrations in the n, p and n^+^ regions are n = 1 × 10^15^/cm^3^, p = 2 × 10^16^/cm^3^ and n^+^ = 5 × 10^19^/cm^3^, respectively. The density of doping is same as [19], while the pattern is different.

As a force sensor, a gold-coated cantilever (Mikromasch: HQ: NSC15/CR-AU) was used. The resonance frequency, spring constant and *Q* value of the cantilever are *f*_res_ = 257.57 kHz, *k* = 40 N/m and *Q* = 10 709, respectively.

To investigate the frequency dependence of the AC bias voltage of *V*_CPD_, the tip–sample distance in both FM-KPFM and heterodyne FM-KPFM measurements must be equal, because the *V*_CPD_ also depends on the tip–sample distance [5–7]. Therefore, we used the multi-pass scanning method in the measurements of FM-KPFM/AFM and heterodyne FM-KPFM/AFM to eliminate the effect of the tip–sample distance dependence. In the first lateral scan, AFM measurement was performed while FM-KPFM measurement was performed. In the second lateral scan, heterodyne FM-KPFM measurement was performed with the trajectory of the first AFM measurement. The frequencies of the AC bias voltage applied between the tip and the sample were *f*_m_ = 100 Hz for FM-KPFM and 2*f*_0_ + *f*_m_ = 515.232 kHz for heterodyne FM-KPFM, respectively. The amplitude of the AC bias voltage was set to *V*_AC_ = 0.5 V to allow comparison of *V*_CPD_ measurements with a high signal-to-noise ratio for both FM-KPFM and heterodyne FM- KPFM. The oscillation amplitude of the cantilever was *A *= 15 nm.

## Results and discussion

**Fig. 5.**
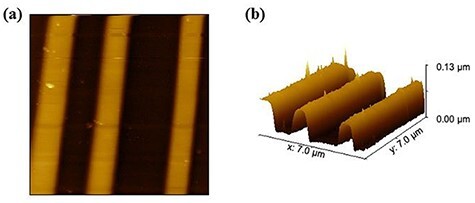
(a) Wide range topographic image on the pn-patterned Si surface by FM-AFM experiment and (b) 3D image. Scan size: 7 μm × 7 μm.

Figure 5a and b shows the topographic image of pn-patterned Si substrate and its 3D image, respectively. By comparing the topographic image in Fig. 5 and the model in Fig. 4, we can see that the surface is higher in the p- and n-type regions than in the n^+^-type region.

**Fig. 6.**
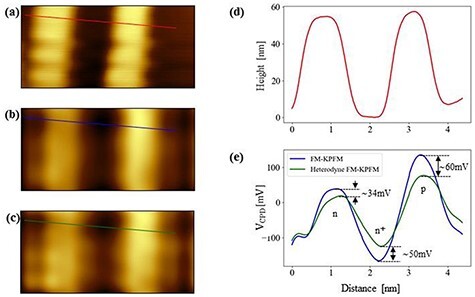
AFM and surface potential images of pn-patterned Si surface using HL-KPFM method. (a) Topographic and (b) *V*_CPD_ images obtained by FM-KPFM with modulation frequency *f*_m_ = 100 Hz. (c) *V*_CPD_ image obtained by heterodyne FM-KPFM with 2*f*_0_ + *f*_m_ ≅ 515.2 kHz. The scan size is 5 μm × 2.36 μm. (d) Line profile along red line in (a). (e) Line profiles along blue line in (b) and along green line in (c). Note that the CPD values are different in the n- and p-type regions. The CPD differences between the n, n^+^ and p regions are due to the Fermi level differences between dopant regions. (n^+^-type region, acceptor-like; n- and p-type regions, donor-like).

Figure 6a and d shows the topographic image measured on a pn-patterned Si substrate and the cross-sectional profile along the red line in Fig. 6a. Figure 6b and c shows CPD images simultaneously obtained by FM-KPFM and heterodyne FM-KPFM, respectively, and Fig. 6e shows the cross-sectional profiles along the blue and green lines in Fig. 6b and c. From the topographic image in Fig. 6a and the line profile in Fig. 6d, we can see that the heights of the p- and n-type regions are almost equal. In contrast, in Fig. 6b, the CPD image by FM-KPFM, the p-type region is brightest, the n-type region is slightly darker and the n^+^-type region is darkest. Similarly, in Fig. 6c, the CPD image by heterodyne FM-KPFM, the p-type region is brightest, the n-type region is slightly darker and the n^+^-type region is darkest. From the line profiles in Fig. 6e, we can see that the V_CPD_ values measured by both FM-KPFM and heterodyne FM-KPFM become lower in the order of the p-type region, n-type region and n^+^-type region. These experimental results are in good agreement with the order of the work functions of pn-patterned Si samples [19].

Interestingly, in Fig. 6e, the line profile of the V_CPD_ values obtained by FM-KPFM is significantly different from those obtained by heterodyne FM-KPFM. In the p- and n-type regions, the V_CPD_ values obtained by heterodyne FM-KPFM become lower than those obtained by FM-KPFM, while in the n+-type region, the V_CPD_ values obtained by heterodyne FM-KPFM become higher than those obtained by FM-KPFM. This is the first result that the V_CPD_ values obtained by KPFM measurement strongly depend on the frequency of the AC bias voltage.

**Fig. 7.**
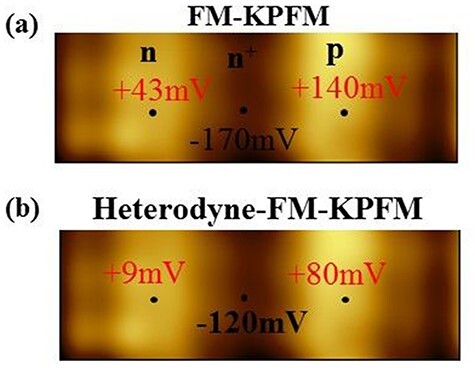
Surface potential image on a pn-patterned Si semiconductor with acceptor-like (n^+^-type region) and donor-like (n- and p-type regions) surface states. (a) V_CPD_ images obtained by FM-KPFM and (b) heterodyne FM-KPFM. Scan size: 5 μm × 1.70 μm. +43 mV on n-type region determined by FM-KPFM (+9 mV: by heterodyne FM-KPFM), −170 mV on n^+^-type region determined by FM-KPFM (−120 mV: by heterodyne FM-KPFM) and +140 mV on p-type region determined by FM-KPFM (+80 mV: by heterodyne FM-KPFM).

Next, we quantitatively investigate the V_CPD_ values in the n-, n^+^- and p-type regions. The V_CPD_ values obtained by FM-KPFM are estimated to be +43 mV, −170 mV and +140 mV near the centers of the n-, n^+^- and p-type regions, respectively (Fig. 7a). The V_CPD_ values obtained by heterodyne FM-KPFM are estimated to be +9 mV, −120 mV and +80 mV near the centers of the n-, n^+^- and p-type regions, respectively (Fig. 7b). Therefore, the V_CPD_ values obtained by heterodyne FM-KPFM varied by −34 mV, +50 mV and −60 mV near the centers of the n-, n^+^- and p-type regions, respectively, compared to those obtained by FM-KPFM. Here, + and − indicate that the band bending occur upwardly and downwardly at the surface, respectively. These experimental results mean that the band bending occur downwardly, upwardly and downwardly, the n-, n^+^- and p-type regions, respectively.

If the surface state created by the broken atomic bonds exists near the center of the band gap, then in the case of an n-type semiconductor, electron transfer occurs from the bulk state to the surface state and the band bending is upward, while in the case of a p-type semiconductor, electron transfer occurs from the surface level to the bulk level and the band bending is downward. The experimental result in Fig. 7 that the band bending is upward in the n^+^-type region and downward in the p-type region is consistent with the above model of electron transfer, but the experimental result that the band bending is downward in the n-type region is not consistent with the above model of electron transfer.

There are two possible causes for the downward band bending in the n-type region: first, many defects may have formed on the sample surface due to ion implantation, and these defects may have formed donor-like surface states; second, a natural oxide film may have formed on the surface, and donor-like interface states may have formed between the natural oxide film and the substrate. In either case, unlike the ideal acceptor-like surface state, a donor-like surface state was formed, and electron transfer from the donor-like surface state to the bulk state may have occurred, causing the band to bend downward.

## Concluding remarks

We performed comparative measurements between FM-KPFM using low frequency bias voltage and heterodyne FM-KPFM using high frequency bias voltage to demonstrate the effectiveness of HL-KPFM on the surface potential measurement. We used a silicon substrate patterned with p- and n-type impurities. We used the multi-pass scanning method in the measurements of FM-KPFM and heterodyne FM-KPFM to eliminate the effect of the tip–sample distance dependence. The measured surface potentials become lower in the order of the p-type region, n-type region and n^+^-type region by both FM-KPFM and heterodyne FM-KPFM, which are in good agreement with the order of the work functions of the pn-patterned Si sample. We observed the difference in the surface potentials measured by FM-KPFM and heterodyne FM-KPFM. The difference is due to the fact that the charge transfer between the surface and bulk levels may or may not respond to AC bias voltage.

In HL-KPFM, it is expected that the bulk and surface states can be more clearly distinguished by using a low frequency bias voltage and a higher frequency bias voltage. Therefore, we believe that the HL-KPFM method can be used to characterize a variety of materials.
